# Total plasma magnesium, zinc, copper and selenium concentrations in type-I and type-II diabetes

**DOI:** 10.1007/s10534-018-00167-z

**Published:** 2019-01-22

**Authors:** Amélie I. S. Sobczak, Fiona Stefanowicz, Samantha J. Pitt, Ramzi A. Ajjan, Alan J. Stewart

**Affiliations:** 1 School of Medicine, University of St Andrews, Medical and Biological Sciences Building, St Andrews, Fife KY16 9TF UK; 2 Scottish Trace Element and Micronutrient Diagnostic and Research Laboratory, Department of Clinical Biochemistry, NHS Greater Glasgow and Clyde, Glasgow, UK; 3 Leeds Institute of Cardiovascular and Metabolic Medicine, University of Leeds, Leeds, UK

**Keywords:** Diabetes, HbA1c, ICP-MS, Metal homeostasis, Zinc/copper ratio

## Abstract

Glycemia and insulin resistance are important regulators of multiple physiological processes and their dysregulation has wide-ranging consequences, including alterations in plasma concentrations of metal micronutrients. Here, magnesium, zinc, copper, selenium and glycated albumin (HbA1c) concentrations and quartile differences were examined in 45 subjects with type-I diabetes (T1DM), 54 subjects with type-II diabetes (T2DM) and 62 control subjects in order to assess potential differences between sexes and between T1DM and T2DM. Plasma magnesium concentration was decreased in T1DM subjects, with the second, third and fourth quartiles of magnesium concentrations associated with the absence of T1DM. This effect was observed in females but not males. In T2DM, the highest quartile of selenium concentrations and the third quartile of copper concentrations associated with the absence of diabetes in males. The highest quartile of magnesium concentrations was associated with the absence of T2DM in males but not females. HbA1c correlated with plasma concentrations of magnesium (negatively, in both sexes together in T1DM and T1DM males), copper (positively, in T1DM males and in both sexes together in T2DM), selenium (positively, in both sexes together in T1DM and T2DM, and T2DM females) and with zinc/copper ratio (negatively, in both sexes together in T1DM and T2DM). This study shows that plasma magnesium concentration is altered to the highest degree in T1DM, while in T2DM, plasma selenium and copper concentrations are significantly affected. This work increases our understanding of how T1DM and T2DM affects plasma metal concentrations and may have future implications for diabetes management.

## Introduction

Diabetes is mainly divided into two types. In type-I diabetes (T1DM), the β-cells of the islets of Langerhans in the pancreas responsible for producing insulin are lost, typically from an attack from the immune system, causing an insulin deficiency in the body. In type-II diabetes (T2DM), cells become resistant to insulin signalling, failing to respond to it sufficiently. This is combined with either normal or increased insulin levels but can also develop into insulin deficiency through a relative loss of the insulin storage function of β-cells. Glucose levels in the body are important, as directly or indirectly they regulate many physiological processes, including glucose, glycogen metabolism. It also acts to control food intake (satiety) and maintaining long-term body weight but also regulates inflammation, vasodilatation and basic cell growth and replication. Thus, mishandling of glucose has wide-ranging consequences in the body. Among them is an alteration in the total plasma concentrations of micronutrients including some metal ions (Kaur and Henry 2014). This is thought to be caused by numerous factors, which include increased fluid loss from the body, an increased demand for micronutrients due to altered protein metabolism as well as defective metal transport due to oxidative stress (Kaur and Henry 2014; Maret 2017).

Variations in plasma metal concentrations in diabetes have been extensively studied in different populations. However there is a great heterogeneity in the data, with both higher and lower levels of the same metals having been reported (e.g. zinc and selenium) (Wang et al. 2016; Sanjeevi et al. 2018). Several meta-analyses have been performed in order to find a consensus on whether levels of some metals are higher or lower in diabetes (Wang et al. 2016; Sanjeevi et al. 2018). Analyses of quartiles of selenium concentrations yielded interesting results, with both the lowest and highest quartiles being associated with T2DM (Wang et al. 2016). Sex differences have also been shown to be of some importance with higher levels of copper reported in females with T1DM but not males (Ruiz et al. 1998). In addition, glycated haemoglobin (HbA1c) level as an indication of diabetes management was found to correlate with several plasma metal concentrations, including zinc, copper and magnesium and also to the zinc/copper ratio (Naka et al. 2013; Ramadass et al. 2015; Atari-Hajipirloo et al. 2016).

Here, we have examined the total plasma concentrations of zinc, copper, magnesium and selenium in patients with T1DM and T2DM and in controls, assessing the correlation between quartiles of metal cations concentrations and diabetes, as well as the influence of sex and diabetes management through blood HbA1c levels. Plasma metal concentrations and potential sex differences in those concentrations have not been thoroughly examined both in T1DM and in T2DM in the same study before. The results indicate that magnesium deficiency is particularly important in T1DM while elevated copper and selenium deficiency in females are more important in T2DM. A better understanding of those effects could lead to a better understanding of metal micronutrient handling and to better management and treatment of T1DM and T2DM.

## Methods

### Samples collection and treatment

A total of 45 patients with T1DM, 54 patients with T2DM and 62 controls were recruited from Leeds Teaching Hospital Trust. Plasma samples from subjects with either T1DM or T2DM and controls were collected following approval by the local Research Ethics Committee in Leeds and after obtaining written informed consent. For all subjects, baseline fasting blood samples were collected in lithium heparin coated tubes. Plasma was then separated within 2 h of collection by centrifugation at 2400×*g* for 20 min at 4 °C. Samples were snap frozen in liquid nitrogen and stored at − 40 °C until analysis.

### Inductively-coupled plasma-mass spectrometry (ICP-MS)

Single element stock solutions (Centripure, Merck, UK) traceable to the United States National Institute of Standards and Technology (NIST) were used for calibration standards and internal standards. All calibration standards were prepared using a solution of 2% v/v butan-1-ol (Sigma Aldrich, USA), 0.05% w/v EDTA (Sigma Aldrich), 0.05% v/v Triton-X-100 (Sigma Aldrich), 1% v/v ammonia (Romil, UK) in 18.2 Mohm reverse osmosis deionised water (Elga Maxima High Wicombe, UK). Lithium heparin plasma samples were diluted 1 in 10 with the same solution used to prepare calibration standards with 25 ppb germanium. ClinChek^®^ 1 & 2 (RECIPE, Germany) and Seronorm™ 1 (SERO, Norway) human serum certified reference materials were used to demonstrate accuracy. ClinChek^®^1 and 2 are materials with consensus values while Seronorm™ is traceable to a National Institute of Standard Technology (NIST) certified reference material.

Copper, zinc, selenium and magnesium analyses were performed simultaneously using an Agilent 7900 ORS-ICP-MS (Agilent Technologies, Santa Clara, California, USA). The instrument was controlled using Mass Hunter software (version 4.1, Agilent Technologies). Argon was used to form the plasma (CryoServices Ltd, purirty: 99.9%). Polyatomic interferences for copper, zinc and magnesium were removed through collision induced dissociation and kinetic energy discrimination using helium gas (Air Products and Chemicals, Inc, purity 99.9%). Polyatomic and doubly charged interferences for selenium were removed using a charge exchange reaction by using the collision cell with hydrogen. The concentration of all elements was measured three times within a single run using the central 0.05 m/z of the peak. The ICP-MS was equipped with an CETAC ASX-500 series autosampler (Teledyne CETAC, Omaha, USA), an Integrated Sample Introduction System and Discrete Sampler—3 (ISIS-DS, Agilent Technologies) and a G32992A re-circulating chiller (Polyscience, Illinois, USA). The ISIS-DS was fitted with a Quartz Scott-Type, Double-Pass Spray Chamber (Agilent Technologies), a glass micromist nebuliser (Agilent Technologies) and a sample loop. The sample loop was prepared in-house using 0.8 mm internal diameter and PTFE sample tubing (Agilent Technologies). A quartz torch with 2.5 mm internal diameter was used (Agilent Technologies). Nickel sampling and skimmer cones were used at all times (Agilent Technologies). All instrument parameters were optimised daily by performing an auto-tune while aspirating a tuning solution containing 10 ppb lithium, yttrium, cobalt, cerium and thallium. Typical instrument parameters are shown in Table 1. Three certified reference materials (Seronorm 1 and ClinCheck 1 and 2) with assigned values and ranges were used to demonstrate the accuracy of the method used to determine the concentrations of Mg, Cu, Zn and Se in human plasma (Table 2). The mean for all measurands were within the assigned range for all materials and had satisfactory recoveries ranging between 96.4 and 107%. The precision of the method, represented by coefficient of variation, was also satisfactory with values less than or equal to 3%. All metal concentrations in plasma were compared to the glycated haemoglobin concentrations which were measured in the samples as a routine test.

**Table 1:** ICP-MS instrument parameters

Parameter	Setting
Isotopes monitored (m/z)	Cu 63, Zn 66, Se 78 and Mg 24
RF power (W)	1550
RF matching	1.70
Sampling depth (mm)	10
Carrier gas (L/min)	1.05
Make up gas L/min)	0.0
Spray chamber temperature (°C)	2
He octopole reaction system flow (mL/min)	5.0
Nebuliser pump (rps)	0.1

**Table 2:** Accuracy of the ICP-MS method and details of the concentration of trace elements in each material (coefficient of variation and recovery)

Measurand	Specimen	Assigned value	Range	Mean	CV (%)	Recovery (%)
Plasma Mg (mM)	ClinCheck 1	0.64	0.58–0.71	0.62	1.8	97.6
	ClinCheck 2	1.21	1.09–1.33	1.17	1.5	96.4
Plasma Cu (μM)	Seronorm1	17.1	15.7–18.7	17.0	1.8	99.6
	ClinCheck 1	10.9	8.7–13.1	10.7	1.3	97.8
	ClinCheck 2	19.1	15.4–23.0	19.0	1.4	98.8
Plasma Zn (μM)	Seronorm1	16.8	14.6–19.0	17.8	3.0	107
	ClinCheck 1	17.7	14.2–21.3	17.6	0.8	99.2
	ClinCheck 2	23.2	18.8–28.3	24.2	1.3	104.2
Plasma Se (μM)	Seronorm1	1.10	0.96–1.25	1.09	0.8	98.8
	ClinCheck 1	1.03	0.82–1.24	1.06	0.7	103
	ClinCheck 2	1.52	1.22–1.82	1.57	1.2	104

### Statistical analysis and representation

Data are presented as mean ± standard error of the mean (SEM). Graphs were generated and statistical analysis was performed using Prism 7.0 (GraphPad Software, La Jolla, CA). Differences between groups were analysed using multiple Student’s t-tests or analysis of variance, while correlations between linear variants were analysed with Pearson’s correlation. The data was also separated into quartiles and odd ratios were calculated. Significance threshold was set at p ≤ 0.05.

## Results and discussion

### Total plasma zinc, copper, magnesium, selenium concentrations and zinc/copper ratios in T1DM and T2DM

ICP-MS was used to determine the total plasma concentrations of zinc, copper, magnesium and selenium in subjects with T1DM or T2DM and in their respective age-matched controls. The zinc/copper ratio was also calculated in all samples. Table 3 summarises the demographic characteristics of our population as well as the mean plasma HbA1c and metal concentrations values in each group. As expected, t-tests indicate that subjects with T1DM or T2DM have higher plasma concentrations of HbA1c than controls. Both diabetes groups also had higher mean BMI values than controls, while the T2DM group also has a higher percentage of male subject than the controls. Mean plasma zinc, copper and selenium concentrations were not significantly altered in subjects with either T1DM or T2DM compared to controls. Whereas the mean plasma magnesium concentration was significantly lower in subjects with T1DM than in controls but not with T2DM, while the plasma zinc/copper ratio was significantly higher in T2DM subjects than in controls, but this was not the case in T1DM.

**Table 3:** Demographic characteristics of the studied population and mean plasma HbA1c and metal concentrations in each group

Characteristics	T1DM subjects (*n *= 45)	T1DM age-matched controls (*n *= 47)	*t* test between T1DM and age-matched controls	T2DM subjects (*n *= 54)	T2DM age-matched controls (*n *= 18)	t-test between T2DM and age-matched controls
Age (years ± SD)	26.3 ± 6.8	24.3 ± 6.2	ns	61.1 ± 7.6	57.1 ± 8.9	ns
Males, *n* (%)	26 (58)	24 (51)	ns	47 (87)	9 (50)	***
BMI						
BMI (kg/m^2^ ± SD)	24.6 ± 3.6	23.0 ± 3.0	*	32.6 ± 5.3	25.0 ± 3.2	***
n (%) for BMI < 25	24 (53)	38 (81)	–	2 (4)	10 (59)	–
n (%) for BMI 25 to < 30	18 (40)	8 (17)	–	17 (31)	6 (35)	–
n (%) for BMI ≥ 30	3 (7)	1 (2)	–	35 (65)	1 (6)	–
HbA1c conc. mean (mM ± SD)	69.8 ± 18.0	33.5 ± 0.7	**	72.4 ± 22.8	37.6 ± 4.2	***
Zinc conc. mean (µM ± SD)	12.80 ± 1.50	13.50 ± 2.30	ns	12.70 ± 1.40	12.38 ± 1.69	ns
Copper conc. mean (µM ± SD)	17.56 ± 6.60	16.26 ± 5.50	ns	15.49 ± 2.78	17.30 ± 4.70	ns
Magnesium conc. mean (mM ± SD)	0.747 ± 0.057	0.806 ± 0.054	***	0.738 ± 0.075	0.765 ± 0.115	ns
Selenium conc. mean (µM ± SD)	1.147 ± 0.145	1.129 ± 0.136	ns	1.142 ± 0.188	1.222 ± 0.261	ns
Zinc/copper (ratio ± SD)	0.813 ± 0.273	0.886 ± 0.246	ns	0.841 ± 0.152	0.751 ± 0.187	*

Significance is indicated as *p < 0.05, **p < 0.01 and ***p < 0.001

The data from each of the two diabetes groups, (T1DM and T2DM subjects; each with their respective age-matched controls) were split into quartiles for each metal, defined as four groups delimited by 25%, median and 75% of highest values in each of the groups. The odd ratios (ORs) of a subject in a specific quartile having diabetes were calculated (Table 4). No significant difference in total plasma zinc concentrations was observed between either the T1DM or the T2DM groups and their age-matched controls. This is contrary to two previous reports—a meta-analysis accumulating 20,183 T2DM subjects and a study with 51 T1DM subjects both showing that zinc concentration is lower in diabetes (Li et al. 2018; Sanjeevi et al. 2018). Despite previous reports of both higher and lower zinc concentrations being associated with T2DM, no significance could be gained by examining quartiles of zinc concentrations (Sanjeevi et al. 2018). In T2DM, the ORs of having diabetes in the second (Q2), third (Q3) and fourth (Q4) copper quartile groups were respectively 0.031 [95% confidence interval (CI) 0.080–1.275, p > 0.05], 0.193 (95% CI 0.051–0.966, p < 0.05) and 0.276 (95% CI 0.070–1.154, p > 0.05) compared with the first (Q1) quartile group. The absence of a clear difference between T2DM subjects and controls (with only Q3 being significantly associated with the absence of diabetes) can be explained by the mean plasma copper concentration being very low in our T2DM group and high in our age-matched controls, almost enough to be significantly higher in the controls (p = 0.0698), whereas a meta-analysis involving 20,183 T2DM individuals has shown that plasma copper is higher in T2DM (Sanjeevi et al. 2018). There were no differences in plasma copper concentrations between quartiles in the T1DM group, contrary to a previous study (Ruiz et al. 1998). The ORs of having T1DM in the Q2, Q3, Q4 magnesium groups were 0.176 (95% CI 0.058–0.628, p < 0.01), 0.1173 (95% CI 0.036–0.432, p < 0.01) and, 0.086 (95% CI 0.028–0.325, p < 0.001) compared with the Q1 group. Thus, T1DM is associated with lower plasma magnesium concentrations (Q2–Q4), as supported by a previous study (Brown et al. 1999). In T2DM, no differences in magnesium concentration were observed between quartiles. This is despite a meta-analysis involving 286,668 T2DM subjects having found an association between low dietary magnesium levels and T2DM, while another study following 12,128 non-diabetic subjects over 6 years reported an inverse relationship between serum magnesium concentrations and incidence of T2DM amongst white (but not black) participants (Kao et al. 1999; Larsson and Wolk 2007). For plasma selenium concentrations, the ORs of having T2DM in the Q2, Q3 and Q4 selenium groups were respectively 0.850 (95% CI 0.221–3.292, p > 0.05), 0.560 (95% CI 0.152–2.484, p > 0.05) and 0.100 (95% CI 0.017–0.682, p < 0.01) compared with the Q1 group. Thus, low selenium concentrations were linked with T2DM. The fact that subjects in Q4 did not associate with T2DM is contrary to the findings of previously published meta-analysis based on 13,460 T2DM patients, where both Q1 and Q4 were associated with T2DM (Wang et al. 2016). No differences in plasma selenium concentrations were observed between quartiles in T1DM, although a previous study indicated that lower plasma selenium concentrations have been found in T1DM (Ruiz et al. 1998). Here we were unable to see any differences between zinc/copper ratio quartiles in either T1DM or T2DM, contrary to the higher mean ratios we measured in T2DM. Some previous publications also indicated that lower plasma zinc/copper ratios have been observed in T1DM (Lin et al. 2014) and in T2DM (Atari-Hajipirloo et al. 2016). The failure to detect some of these previously reported relationships in our study may be due to the number of subjects (and the resultant effect on statistical power). Thus larger cohort studies enable relatively subtle differences in plasma metal concentrations to be observed between individuals with diabetes and controls. However, those differences may not be as relevant as those that can be detected in smaller cohorts.

**Table 4:** Prevalences of diabetes in  quartiles of total plasma concentrations of zinc, copper, magnesium and selenium and zinc/copper ratio in T1DM and T2DM study groups (including age-matched controls)

	T1DM and age-matched controls				T2DM and age-matched controls			
	Q1	Q2	Q3	Q4	Q1	Q2	Q3	Q4
Zinc (µM)	n = 23	n = 23	n = 30	n = 16	n = 15	n = 16	n = 20	n = 20
	< 11.95	11.95–12.91	12.91–14.46	≥ 14.46	< 11.33	11.33–12.15	12.15–13.35	≥ 13.35
% diabetes	52	48	60	25	73	75	75	80
OR (95% CI)	1	0.840 (0.285–2.873)	1.375 (0.457–4.234)	0.306 (0.090–1.204)	1	1.091 (0.264–4.490)	1.091 (0.281–5.312)	1.455 (0.357–5.822)
Copper (µM)	n = 22	n = 17	n = 23	n = 26	n = 33	n = 13	n = 12	n = 12
	< 13.99	13.99–14.47	14.47–16.79	≥ 16.79	< 15.06	15.06–16.40	16.40–17.78	≥ 17.78
% diabetes	50	29	48	64	88	69	58	67
OR (95% CI)	1	0.417 (0.111–1.541)	0.9167 (0.310–2.790)	1.800 (0.559–5.139)	1	0.310 (0.080–1.275)	0.193 (0.051–0.966)*	0.276 (0.070–1.154)
Magnesium (mM)	n = 41	n = 17	n = 15	n = 19	n = 23	n = 25	n = 13	n = 9
	< 0.78	0.78–0.81	0.81–0.83	≥ 0.83	< 0.72	0.72–0.79	0.79–0.84	≥ 0.84
% diabetes	75	35	27	21	83	84	69	44
OR (95% CI)	1	0.176 (0.058–0.628)**	0.117 (0.036–0.432)**	0.086 (0.028–0.325)***	1	1.105 (0.288–4.220)	0.474 (0.118–1.969)	0.168 (0.031–1.064)
Selenium (µM)	n = 21	n = 20	n = 19	n = 32	n = 24	n = 21	n = 19	n = 6
	< 1.04	1.04–1.12	1.12–1.18	≥ 1.18	< 1.085	1.09–1.24	1.24–1.42	≥ 1.42
% diabetes	52	40	37	59	83	80	74	33
OR (95% CI)	1	0.606 (0.172–1.958)	0.530 (0.136–1.784)	1.329 (0.424–4.188)	1	0.850 (0.221–3.292)	0.560 (0.152–2.484)	0.100 (0.017–0.682)*
Zinc/copper	n = 29	n = 23	n = 19	n = 20	n = 9	n = 35	n = 25	n = 27
	< 0.734	0.734–0.893	0.893–1.070	≥ 1.070	< 0.667	0.667–0.732	0.732–0.865	≥ 0.865
% diabetes	62	48	37	45	56	86	80	85
OR (95% CI)	1	0.560 (0.172–1.695)	0.357 (0.104–1.233)	0.500 (0.169–1.660)	1	4.800 (1.110–19.940)	3.200 (0.732–13.560)	4.600 (1.001–22.260)

Data is presented as OR (95% CI)

Significance is indicated as *p < 0.05, **p < 0.01 and ***p < 0.001

### Differences in plasma metal concentrations between males and females with T1DM or T2DM

The influence of the sex of the subjects on plasma metal concentrations between the various groups was examined (Table 5). In our age-matched controls for the T1DM group, males had significantly higher zinc concentrations and lower copper concentrations than females, while in the T1DM group males had significantly higher zinc and magnesium concentrations and lower copper concentrations than females. The T2DM group was harder to study as there were few females in the group. However, copper concentrations were significantly lower and the zinc/copper ratio higher in males than in females. The T1DM and T2DM groups were then compared with their respective age-matched controls. Magnesium concentrations were lower in males and females with T1DM than in the respective controls, while only males with T2DM had a lower magnesium concentration than their respective controls. In addition, the zinc/copper ratio was higher in males with T2DM than in the male controls.

**Table 5:** Differences in total plasma concentrations of zinc, copper, magnesium and selenium and zinc/copper ratio in males and females with T1DM and T2DM (and respective age-matched controls)

Parameters (mean ± SD)	Controls age-match with T1DM			T1DM			Differences between T1DM and age-matched controls	
	Males (n = 24), mean ± SD	Females (n = 23), mean ± SD	t-test between male and female controls	Males (n = 26), mean ± SD	Females (n = 19), mean ± SD	t-test between males and females with T1DM	t-test between T1DM males and control males	t-test between T1DM females and control females
Zinc (µM)	14.34 ± 2.72	12.62 ± 1.33	**	13.22 ± 1.538	12.22 ± 1.236	*	ns	ns
Copper (µM)	13.70 ± 1.44	18.81 ± 6.79	***	14.88 ± 3.727	21.23 ± 7.911	***	ns	ns
Magnesium (mM)	0.807 ± 0.056	0.804 ± 0.054	ns	0.763 ± 0.056	0.726 ± 0.052	*	**	***
Selenium (µM)	1.151 ± 0.117	1.106 ± 0.152	ns	1.120 ± 0.143	1.184 ± 0.145	ns	ns	ns
Zinc/copper ratio	1.025 ± 0.139	0.747 ± 0.253	***	0.930 ± 0.220	0.651 ± 0.260	***	ns	ns

Parameters (mean ± SD)	Controls age-match with T2DM			T2DM			Differences between T2DM and age-matched controls	
	Males (n = 9), mean ± SD	Females (n = 8), mean ± SD	t-test between male and female controls	Males (n = 46), mean ± SD	Females (n = 7), mean ± SD	t-test between males and females with T2DM	t-test between T2DM males and control males	t-test between T2DM females and control females
Zinc (µM)	12.33 ± 1.35	12.43 ± 2.11	ns	12.72 ± 1.45	12.32 ± 1.46	ns	ns	ns
Copper (µM)	16.44 ± 1.53	18.27 ± 6.76	ns	15.12 ± 2.28	18.67 ± 1.31	**	ns	ns
Magnesium (mM)	0.812 ± 0.083	0.712 ± 0.128	ns	0.737 ± 0.078	0.757 ± 0.047	ns	*	ns
Selenium (µM)	1.273 ± 0.149	1.165 ± 0.351	ns	1.153 ± 0.179	1.070 ± 0.229	ns	ns	ns
Zinc/copper ratio	0.757 ± 0.108	0.744 ± 0.257	ns	0.865 ± 0.140	0.683 ± 0.141	**	*	ns

Significance is indicated as *p < 0.05, **p < 0.01 and ***p < 0.001

The plasma metal concentration quartile groups described above were split according to sex (Table 6). The ORs of having T1DM in the Q2, Q3 and Q4 female magnesium quartile groups were respectively 0.000 (95% CI 0–0.480, p < 0.01), 0.059 (95% CI 0.005–0.482, p < 0.05) and 0.033 (95% CI 0.003–0.287, p < 0.001) compared to the Q1 group. There were no differences between magnesium quartiles in T1DM males despite a lower mean having been observed compared to male controls. To our knowledge, no sex differences in mean plasma magnesium concentrations in T1DM subjects have been reported before. No significance differences in male copper quartiles or in female copper quartiles were observed in T1DM. This is contrary to a report, which found that plasma copper concentrations were higher in females with T1DM than in females controls but that this was not the case in males (Ruiz et al. 1998). However another study (with only 18 individuals and both T1DM and T2DM analysed together) reported the absence of any difference in plasma copper concentration between males and females with diabetes (Terres-Martos et al. 1998). In addition, no significant differences in male or in female zinc or zinc/copper quartiles were identified in T1DM.

**Table 6:** Prevalences of diabetes in quartiles of total plasma zinc, copper, magnesium and selenium concentration and zinc/copper ratio seperated by sex in T1DM and T2DM study groups (including age-matched controls)

		T1DM and age-matched controls				T2DM and age-matched controls			
		Q**1**	Q2	Q3	Q4	Q1	Q2	Q3	Q4
Zinc (µM)		11.95	11.95–12.91	12.91–14.46	≥ 14.46	< 11.33	11.33–12.15	12.15–13.35	≥ 13.35
Males	n	10	9	17	14	11	11	16	16
	OR (95% CI)	1	0.533 (0.078–2.843)	1.600 (0.370–7.145)	0.267 (0.047–1.600)	1	2.222 (0.221–35.010)	0.667 (0.110–3.837)	3.333 (0.334–51.000)
Females	n	13	14	13	2	3	5	4	4
	OR (95% CI)	1	1.167 (0.261–5.488)	1.000 (0.203–4.927)	0.000 (0.000–3.184)	1	1.333 (0.091–28.020)	4.000 (0.175–83.060)	0.667 (0.027–18.510)
Copper (µM)		< 13.99	13.99–14.47	14.47–16.79	≥ 16.79	< 15.06	15.06–16.40	16.40–17.78	≥ 17.78
Males	n	18	14	11	6	28	11	9	6
	OR (95% CI)	1	0.320 (0.088–1.558)	1.400 (0.315–5.436)	4.000 (0.514–52.23)	1	0.167 (0.011–1.649)	0.074 (0.006–0.625)*	0.074 (0.005–0.842)
Females	n	4	3	12	23	5	2	3	6
	OR (95% CI)	1	1.500 (0.054–37.03)	1.500 (0.165–23.84)	3.900 (0.490–53.94)	1	0.000 (0.000–5.724)	0.750 (0.036–11.030)	3.000 (0.321–26.480)
Magnesium (mM)		< 0.78	0.78–0.81	0.81–0.83	≥ 0.83	< 0.72	0.72–0.79	0.79–0.84	≥ 0.84
Males	n	19	13	9	9	19	18	9	8
	OR (95% CI)	1	0.306 (0.074–1.226)	0.179 (0.040 to 1.033)	0.179 (0.040–1.033)	1	0.944 (0.047–18.900)	0.194 (0.013–2.000)	0.056 (0.004–0.582)*
Females	n	22	4	6	10	4	7	4	1
	OR (95% CI)	1	0.000 (0.000–0.480)**	0.059 (0.005–0.482)*	0.033 (0.003–0.287)***	1	4.000 (0.359–63.030)	3.000 (0.194–56.040)	0.000 (0.000–36.000)
Selenium (µM)		< 1.04	1.04–1.12	1.12–1.18	≥ 1.18	< 1.085	1.09–1.24	1.24–1.42	≥ 1.42
Males	n	12	10	12	16	17	16	18	3
	OR (95% CI)	1	0.750 (0.144–3.807)	0.250 (0.045–1.383)	0.500 (0.129–2.201)	1	0.938 (0.047–18.890)	0.2188 (0.01698–1.703)	0.031 (0.002–0.601)*
Females	n	9	10	7	16	7	5	1	3
	OR (95% CI)	1	0.500 (0.074–3.211)	1.500 (0.240–9.071)	4.400 (0.722–20.100)	1	0.500 (0.061–4.077)	0.000 (0.000–9.000)	0.375 (0.021–4.924)
Zinc/copper		< 0.734	0.734–0.893	0.893–1.070	≥ 1.070	< 0.667	0.667–0.732	0.732–0.865	≥ 0.865
Males	n	4	14	14	17	4	7	21	24
	OR (95% CI)	1	0.000 (0.000–2.397)	0.000 (0.000–1.316)	0.000 (0.000–1.408)	1	1.333 (0.138–12.360)	6 (0.662–45.600)	11 (1.069–89.650)
Females	n	25	9	5	3	5	3	4	3
	OR (95% CI)	1	0.393 (0.094–1.827)	0.196 (0.015–1.616)	0.393 (0.026–3.839)	1	0.333 (0.018–5.167)	0.667 (0.062–7.580)	0.333 (0.018–5.166)

Data is presented as OR (95% CI)

Significance is indicated as *p < 0.05, **p < 0.01 and ***p < 0.001

The low number of females among our T2DM cohort made accurate comparison between the sexes more difficult to perform and no statistically significant differences were found with any metal when examining female subjects in the quartiles. However, when looking at males only the copper Q3, magnesium Q4 and selenium Q4 groups were associated with the absence of T2DM. The ORs of having T2DM in the Q2, Q3 and Q4 male copper quartile groups were respectively 0.167 (95% CI 0.011–1.649, p > 0.05), 0.074 (95% CI 0.006–0.625, p < 0.05) and 0.074 (95% CI 0.005–0.842, p > 0.05) compared to the Q1 group. In a meta-analysis of copper concentrations in T2DM, no sex differences were observed (Sanjeevi et al. 2018). The ORs of having T2DM in the Q2, Q3 and Q4 male magnesium quartile groups were respectively 0.9444 (95% CI 0.04734–18.9, p > 0.05), 0.1944 (95% CI 0.01285–2, p > 0.05) and 0.056 (95% CI 0.004–0.582, p < 0.05) compared to the Q1 group. Contrary to this, a previous study has found that females with T2DM were more likely to have lower magnesium concentrations than males (Kao et al. 1999). The ORs of having T2DM in the Q2, Q3 and Q4 male selenium quartile groups were respectively for selenium 0.938 (95% CI 0.047–18.89, p > 0.05), 0.219 (95% CI 0.017–1.703, p > 0.05), 0.031 (95% CI 0.002–0.601, p < 0.05) compared to the Q1 group. Association between T2DM and selenium concentrations (too low or too high) in males but not females have been reported before (Bleys et al. 2007; Laclaustra et al. 2009; Akbaraly et al. 2010).

### Relationship between plasma metal concentrations and HbA1c concentration

**Fig. 1:**
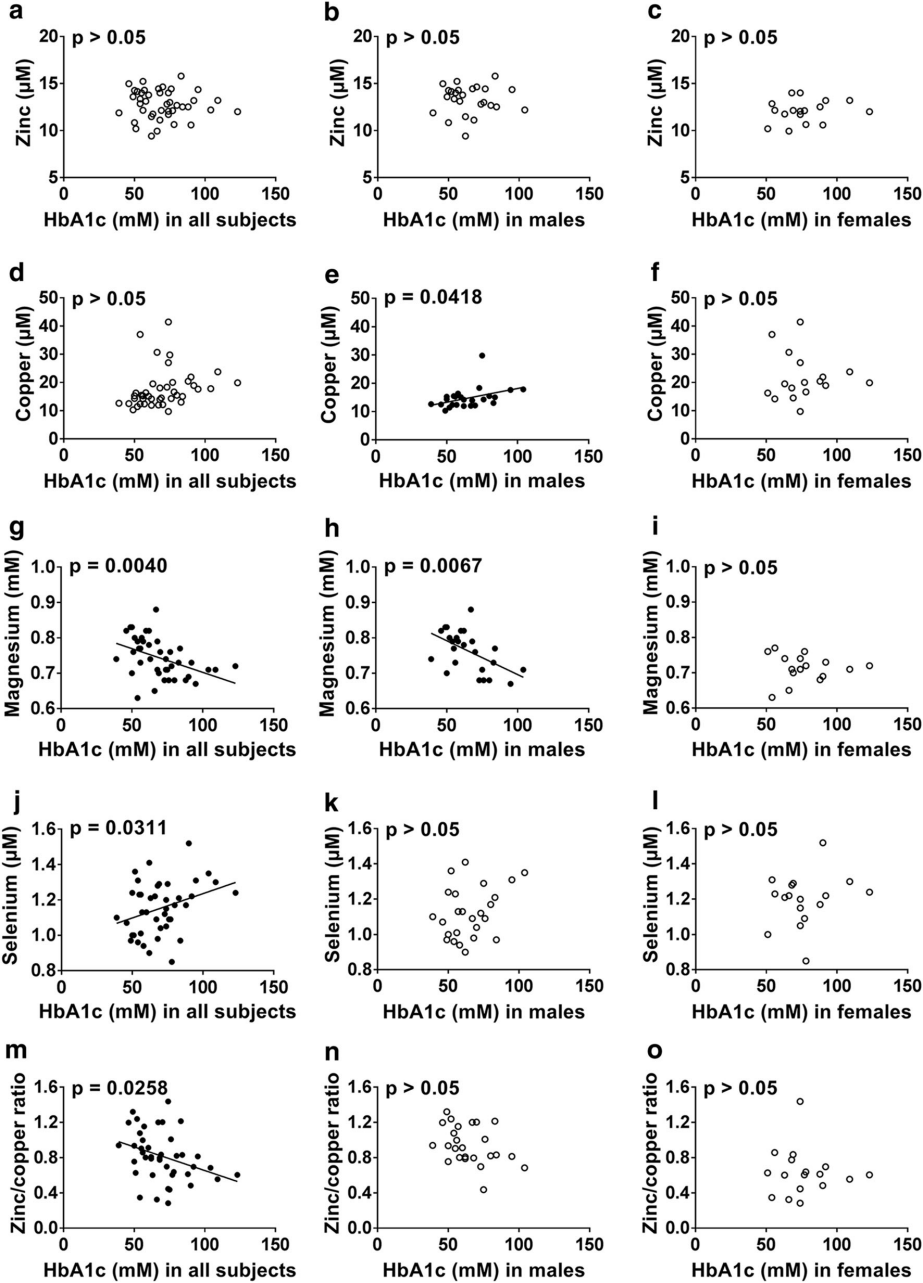
Relationship between HbA1c concentration and plasma zinc, copper, magnesium and selenium concentrations as well as zinc/copper ratio in all T1DM subjects and male and female T1DM subjects. **a**–**c** HbA1c concentration vs zinc concentration, **d**–**f** HbA1c concentration vs copper concentration, **g**–**i** HbA1c concentration vs magnesium concentration, **j**–**l** HbA1c concentration vs selenium concentration and **m**–**o** HbA1c concentration vs zinc/copper ratio. **a**, **d**, **g**, **j**, **m** both sexes together, **b**, **e**, **h**, **k**, **n** male subjects and **c**, **f**, **i**, **l**, **o** female subjects. Black circles were used for the data that were correlated to HbA1c concentration, while white circles were used when the relationship was not significant. HbA1c concentration was positively correlated with plasma copper concentration in males (p = 0.0418) and with plasma selenium concentration in both sexes together (p = 0.0311), while it was negatively correlated with plasma magnesium concentration in both sexes together (p = 0.0040) and in males (p = 0.0067) and with the zinc/copper ratio in both sexes together (p = 0.0258)

**Fig. 2:**
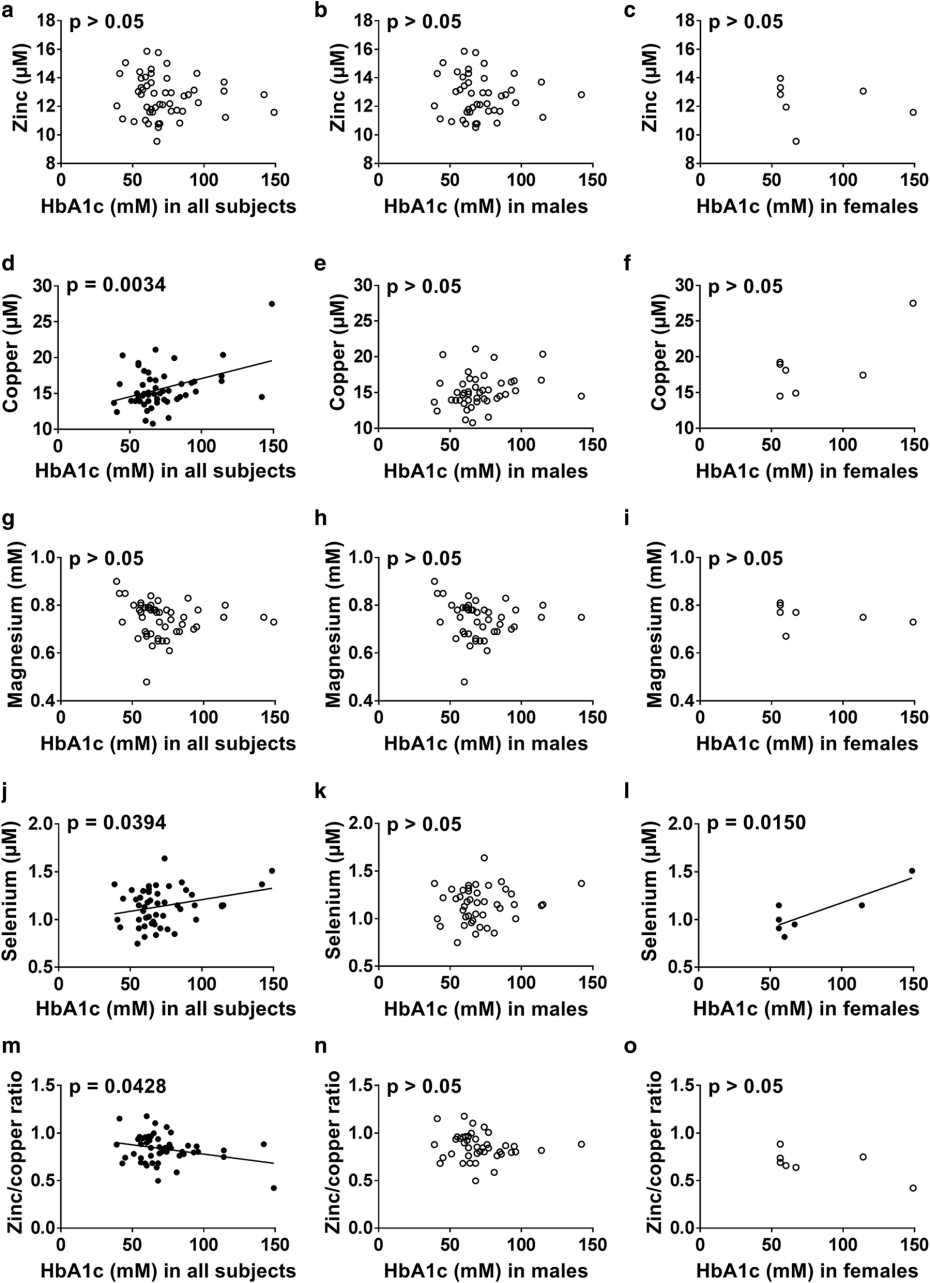
Relationship between HbA1c concentration and plasma zinc, copper, magnesium and selenium concentrations as well as zinc/copper ratio in all T2DM subjects and male and female T2DM subjects. **a**–**c** HbA1c concentration vs zinc concentration, **d**–**f** HbA1c concentration vs copper concentration, **g**–**i** HbA1c concentration vs magnesium concentration, **j**–**l** HbA1c concentration vs selenium concentration and **m**–**o** HbA1c concentration vs zinc/copper ratio. **a**, **d**, **g**, **j**, **m** both sexes together, **b**, **e**, **h**, **k**, **n** male subjects and **c**, **f**, **i**, **l**, **o** female subjects. Black circles were used for the data that were correlated to HbA1c concentration, while white circles were used when the relationship was not significant. HbA1c concentration was positively correlated with plasma copper concentration in both sexes together (p = 0.0034) and with plasma selenium concentration in both sexes together (p = 0.0394) and in females (p = 0.0150), while it was negatively correlated with the zinc/copper ratio in both sexes together (p = 0.0428)

Relationships between HbA1c concentration, as a proxy for glycemic control, and metal concentrations in subjects with T1DM (Fig. 1) or T2DM (Fig. 2) were examined. Plasma zinc concentration did not correlate with HbA1c concentration in either T1DM or T2DM, which is in accord with a previous finding in T1DM but contrary to a previous publication that has found a negative correlation (Luo et al. 2015; Lin et al. 2016). In T1DM, plasma copper concentration positively correlated with HbA1c concentration in males (p = 0.042, Fig. 1e) but not with females or with both sexes together. In T2DM, the plasma copper concentration correlated with HbA1c concentration only when considering both sexes together (p = 0.003, Fig. 2d) but not in males or females only. Previous studies have found that plasma copper concentration do not correlate with HbA1c concentration in T1DM but positively correlates in T2DM (Ruiz et al. 1998; Atari-Hajipirloo et al. 2016). Plasma magnesium concentration negatively correlated with HbA1c concentration in T1DM when looking at both sexes together (p = 0.004, Fig. 1g) and in males (p = 0.007, Fig. 1h), but not in females and it did not correlate with HbA1c concentration in T2DM. The lack of an observable relationship between plasma magnesium concentration and HbA1c concentration in females with T1DM is surprising considering that magnesium concentration was lower in females with T1DM compared to males with T1DM (while no difference between the sexes could be seen in controls). Previous studies have shown that HbA1c levels correlate with magnesium excretion in females but not males (Brown et al. 1999; Lin and Huang 2015). A negative correlation between plasma magnesium concentration and HbA1c concentration has also been reported in T2DM (Ramadass et al. 2015). Plasma selenium concentration correlated negatively with HbA1c in T1DM when looking at both sexes together (p = 0.031, Fig. 1j), as in a previous study (Ruiz et al. 1998), but there was no correlation when looking individually at males or females. In T2DM, plasma selenium concentration was correlated with HbA1c concentration when looking at both sexes together (p = 0.039, Fig. 2j) and in females (p = 0.015, Fig. 2l), contrary to a previous study that found a negative correlation with dysregulation of glucose and selenium concentration in males but not in females (Akbaraly et al. 2010). The plasma zinc/copper ratio was negatively correlated with HbA1c in T1DM and T2DM when looking at both sexes together (p = 0.0258, Fig. 1m and p = 0.0428, Fig. 2m respectively) but not in males or females only. In previous publications, the zinc/copper ratio was also negatively correlated in T1DM and T2DM (Lin et al. 2014; Atari-Hajipirloo et al. 2016). Correlation of HbA1c concentration with total plasma metal concentrations could potentially be explained by glycation or oxidation-associated modifications of metal ion transport proteins (Abdelmagid et al. 2015), formation of glycocholate (anion of the bile acid glycholic acid) that can complex and excrete metals in bile (Atari-Hajipirloo et al. 2016) or abnormal insulin signalling leading to dysregulated metal homeostasis (McNair et al. 1982; Gommers et al. 2016).

### Implications for dysregulated metal homeostasis in diabetes

Changes in total plasma metal concentrations in subjects with diabetes have the potential to negatively affect metabolic processes in the body. Low magnesium concentrations have been associated with an increased incidence of T1DM and T2DM and poor glycaemic control (Lin and Huang 2015; Ramadass et al. 2015). This may be due to the fact that magnesium ions (Mg^2+^) are an essential cofactor in several processes. Mg^2+^ increases the affinity of insulin receptors for ATP and is thus essential for their auto-phosphorylation and tyrosine kinase activity, which results in Mg^2+^ sensitising cells to insulin (Guerrero-Romero and Rodriguez-Moran 2011; Gommers et al. 2016; Al Alawi et al. 2018). Thus chronically low Mg^2+^ concentrations can lead to insulin resistance (Guerrero-Romero and Rodriguez-Moran 2011; Gommers et al. 2016; Al Alawi et al. 2018). Furthermore, Mg^2+^ can also block the entry of Ca^2+^ into adipocytes through the L-type calcium channel. Therefore, when Mg^2+^ concentrations are insufficient, increased Ca^2+^ entry in adipocyte leads to inflammation, oxidative stress and increase insulin resistance (Nielsen et al. 2007; Gommers et al. 2016; Al Alawi et al. 2018). Mg^2+^ is also involved in the transport of glucose across membranes, it is a cofactor of several enzymes essential for carbohydrate oxidation (e.g. phosphotransferases and phosphohydrolases such as ATPases) and also plays a role in the release of insulin (Saris et al. 2000; Al Alawi et al. 2018). On the other hand, insulin decreases tubular reabsorption of Mg^2+^ and so high insulin levels (as in T2DM) could lead to reduced Mg^2+^ concentrations (McNair et al. 1982; Gommers et al. 2016).

Selenium is mostly found in selenoproteins (as selenomethionine), of which many are important anti-oxidants (Roman et al. 2014). Therefore, high plasma concentrations of selenium may be due to oxidative stress mobilising high levels of selenoproteins. For example, the plasma concentration of selenoprotein P is known to be higher in T2DM subjects, a disease state associate with high levels of oxidative stress, and to be associated with insulin resistance (Misu et al. 2010; Ogawa-Wong et al. 2016). However, expression of plasma glutathione peroxidase (another selenoprotein) as well as serum albumin (which transports selenomethionine) are reduced in diabetes (Roman et al. 2010). Expression of selenoprotein P is known to be reduced during inflammation (Nichol et al. 1998; Hesse-Bahr et al. 2000). Thus blood levels of selenium decrease during systemic inflammatory responses, which occur in T2DM (Wang et al. 2013). It has been hypothesised that the stages of the disease (impaired glucose tolerance or well-established T2DM) and the associated level of oxidative stress influences plasma selenium concentration (Rayman and Stranges 2013).

Zinc participates in the synthesis, storage and release of insulin. Notably, zinc is present in secretory vesicles within β-cells of the pancreas where it participates in the crystallisation/storage of insulin and is thus released alongside insulin into the plasma (Scott 1934; Chabosseau and Rutter 2016). Deficiency of zinc disrupts insulin homeostasis, resulting in a reduction of insulin secretion by β cells (Fung et al. 2015). Zinc also stimulates lipogenesis and glucose uptake and reduced lipolysis in adipocytes (Coulston and Dandona 1980; Nishide et al. 2008). As zinc and copper homeostasis are closely linked, notably through competition during intestinal absorption and through shared transporter proteins such as serum albumin, a deficiency in one can affect the other (Osredkar and Sustar 2011). For example, zinc over-supplementation can lead to copper deficiency (Duncan et al. 2015). Impaired metabolism of zinc and copper are associated with a higher sensitivity to oxidative damage, as both zinc and copper are needed for the activity of the antioxidant enzyme superoxide dismutase, whose activity is reduced in T2DM (Sundaram et al. 1996). Thus, changes in the ratio of zinc/copper will affect enzyme catalysis and potentially further increase levels of free radicals, which are already increased in diabetes (Giacco and Brownlee 2010). In addition, copper is released from superoxide dismutase at high glucose concentrations due to fragmentation of the protein during glycation (Ookawara et al. 1992). Elevated copper levels in T2DM correlate with formation of reactive oxygen species (Masad et al. 2007). Insulin also reduces copper concentration in the liver through the regulation of at least one copper-transporting ATPase, ATP7B (Hilario-Souza et al. 2016). A reduction in insulin concentrations may thus result in an accumulation of copper in the liver.

Deficiency in magnesium, selenium and zinc have been shown to increase the risks of developing complicates associated with diabetes. Therefore, the effect of supplementation of these metals has been assessed. Magnesium supplementation was found to both improve diabetes management and to reduce the development of T2DM in individuals at risk (Rodriguez-Moran and Guerrero-Romero 2003; Hruby et al. 2014; Guerrero-Romero et al. 2015). Zinc supplementation has been shown to improve insulin and glucose levels in diabetes subjects and decrease the risk of developing T2DM (Jayawardena et al. 2012; Islam et al. 2016; Ranasinghe et al. 2018). However, selenium supplementation was not found to improve the risk of developing T2DM or the risk of developing complications in individuals with diabetes (Ogawa-Wong et al. 2016). This could be because the association between selenium concentrations and diabetes is not linear and that only individuals with selenium deficiency would benefit from supplementation, while individuals with sufficient selenium could increase their risk of developing T2DM if given an excess of this metal (Ogawa-Wong et al. 2016). Another explanation may be that the form of selenium used for supplementation in some studies offer variable bioavailability (Ogawa-Wong et al. 2016).

## Conclusion

In conclusion, the study reported here is the first to examine zinc, copper, magnesium, selenium and HbA1c concentrations and sex-specific differences together in both T1DM, T2DM and controls. The homeostatic control of circulatory metal concentrations, in particular zinc, copper, magnesium and selenium, is essential for insulin regulation and energy metabolism. If plasma metal concentrations are altered in an individual with diabetes, the management of their condition will be more complex and they are more likely to develop complications. In addition, plasma metal concentrations are also influenced by insulin and glucose plasma levels. Thus, if those are not properly controlled, plasma metal concentrations are likely to become further dysregulated. Here we show that plasma magnesium concentration is altered to the highest degree in T1DM. In T2DM, plasma selenium and copper concentrations were significantly affected. This work increases our understanding of T1DM and T2DM pathogenesis and may have future implications for the management of diabetes.

## Abbreviations

CI: Confidence interval
HbA1c: Glycated haemoglobin
ICP-MS: Inductively-coupled plasma-mass spectrometry
OR: Odds ratio
Q: Quartile
T1DM: Type-I diabetes mellitus
T2DM: Type-II diabetes mellitus
